# Influence of Thin Slice Reconstruction on CT Brain Perfusion Analysis

**DOI:** 10.1371/journal.pone.0137766

**Published:** 2015-09-11

**Authors:** Edwin Bennink, Jaap Oosterbroek, Alexander D. Horsch, Jan Willem Dankbaar, Birgitta K. Velthuis, Max A. Viergever, Hugo W. A. M. de Jong

**Affiliations:** 1 Department of Radiology, University Medical Center Utrecht, Utrecht, the Netherlands; 2 Image Sciences Institute, University Medical Center Utrecht, Utrecht, the Netherlands; INSERM U894, FRANCE

## Abstract

**Objectives:**

Although CT scanners generally allow dynamic acquisition of thin slices (1 mm), thick slice (≥5 mm) reconstruction is commonly used for stroke imaging to reduce data, processing time, and noise level. Thin slice CT perfusion (CTP) reconstruction may suffer less from partial volume effects, and thus yield more accurate quantitative results with increased resolution. Before thin slice protocols are to be introduced clinically, it needs to be ensured that this does not affect overall CTP constancy. We studied the influence of thin slice reconstruction on average perfusion values by comparing it with standard thick slice reconstruction.

**Materials and Methods:**

From 50 patient studies, absolute and relative hemisphere averaged estimates of cerebral blood volume (CBV), cerebral blood flow (CBF), mean transit time (MTT), and permeability-surface area product (PS) were analyzed using 0.8, 2.4, 4.8, and 9.6 mm slice reconstructions. Specifically, the influence of Gaussian and bilateral filtering, the arterial input function (AIF), and motion correction on the perfusion values was investigated.

**Results:**

Bilateral filtering gave noise levels comparable to isotropic Gaussian filtering, with less partial volume effects. Absolute CBF, CBV and PS were 22%, 14% and 46% lower with 0.8 mm than with 4.8 mm slices. If the AIF and motion correction were based on thin slices prior to reconstruction of thicker slices, these differences reduced to 3%, 4% and 3%. The effect of slice thickness on relative values was very small.

**Conclusions:**

This study shows that thin slice reconstruction for CTP with unaltered acquisition protocol gives relative perfusion values without clinically relevant bias. It does however affect absolute perfusion values, of which CBF and CBV are most sensitive. Partial volume effects in large arteries and veins lead to overestimation of these values. The effects of reconstruction slice thickness should be taken into account when absolute perfusion values are used for clinical decision making.

## Introduction

**Fig 1:**
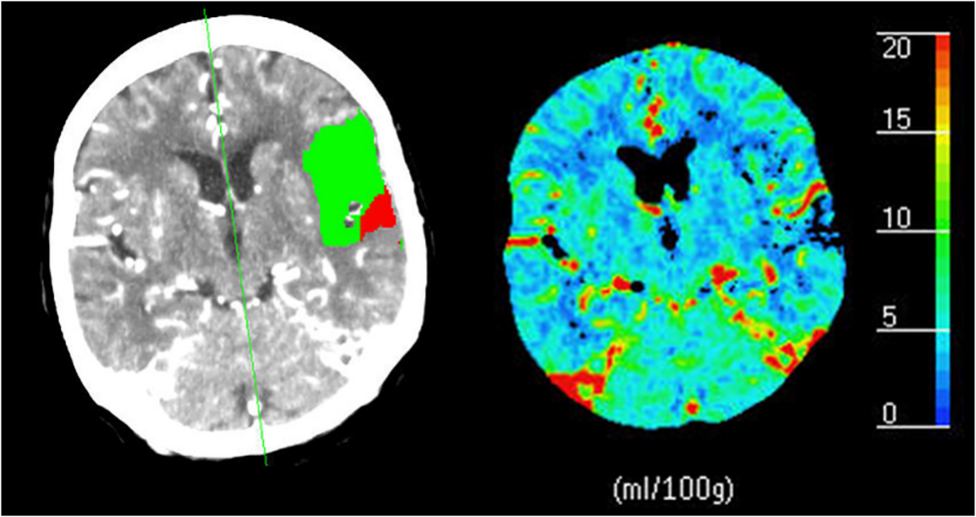
A typical CT perfusion summary map and blood volume map. An example of a thick slice (5 mm) CTP summary map (left) for acute stroke assessment of a patient with an occluded left middle cerebral artery. The red area indicates the infarct core and the green area indicates the penumbra (tissue at risk). The right parameter map shows the cerebral blood volume (CBV). Both maps were generated by the Philips EBW 4.0 Brain Perfusion software (Philips Healthcare, Best, The Netherlands). The thresholds for the infarct core and penumbra were respectively CBV < 2.0 mL/100g and MTT > 145% of the contralateral side, as suggested by Wintermark et al. [**11**].

CT perfusion (CTP) imaging, or dynamic contrast enhanced CT (DCE-CT), is of interest as a diagnostic and prognostic tool in acute stroke imaging, since it may aid in treatment decision making and outcome prediction [1]. Imaging a contrast bolus passage through cerebral tissue by a series of CT images allows the estimation of several perfusion parameters like cerebral blood volume (CBV), cerebral blood flow (CBF), mean transit time (MTT), and blood-brain barrier permeability. The first three parameter maps may provide insight into which perfusion areas are healthy, at risk for infarction (penumbra), or already infarcted (infarct core). Many studies have presented absolute, relative, or multi-variate thresholds on the perfusion parameters for the classification of these areas [2–12]. For the assessment of acute stroke these areas are often presented in a so-called summary map as shown in Fig 1. The permeability-surface area product (PS) quantifies blood-brain-barrier damage, which is of interest as it is thought to be a predictor for the development of hemorrhagic transformation [13,14].

Modern multi-detector row CT scanners typically have a focal spot size of about 1 mm, an in-plane voxel size of about 0.5 mm × 0.5 mm, and a minimal slice thickness between 0.5 and 1 mm. In clinical practice, however, the CTP brain scans are acquired in thin slices but reconstructed into thicker slices of approximately 5 or 10 mm to suppress noise and to reduce the amount of data and processing time. For this reason thick slice data is generally used to classify infarct core and penumbra. All studies referenced above used a thickness of 5 mm or more, resulting in anisotropic voxel dimensions. Because of ongoing developments in computer performance, it may now be feasible to exploit thin slice CTP brain data with smaller, less anisotropic voxels, which could have substantial benefits in clinical stroke assessment.

First, a slice thickness of 1.8 mm and smaller permits the selection of a partial volume free arterial input function (AIF) in the middle cerebral artery, thus avoiding the necessity for selecting a venous output function (VOF) to correct for partial volume effects (PVE) [15]. It is hypothesized that a significant proportion of the variability found between observers and between analysis platforms in absolute quantification of perfusion values [6,16,17] may be due to the selection of partial volume affected arteries and veins.

Second, because the voxels in thin slice scans are near isotropic, it can be considered a true 3D volume instead of a series of 2D slices. This enables true 3D motion correction as well as sagittal, coronal, and oblique reformatting of perfusion maps.

Third, it has been suggested that the detectability of lacunar infarcts may improve with increased spatial resolution [18]. This type of infarct, generally considered smaller than 15 mm, accounts for 25% of all ischemic strokes [19].

Since modern scanners, computers, and filtering techniques make CTP analysis with high axial resolution feasible, the clinical application of thin slice reconstruction is expected to increase in the near future. Whereas it is known that PVE due to thick slice reconstruction results in overestimation of CBV and CBF [20], the extent of using full axial resolution on perfusion values and outcomes is as yet unclear.

Accordingly, the aim of this study was to assess the consequences of changing the slice thickness of CTP reconstruction on the perfusion parameters measured with CTP brain analysis.

## Materials and Methods

**Fig 2:**
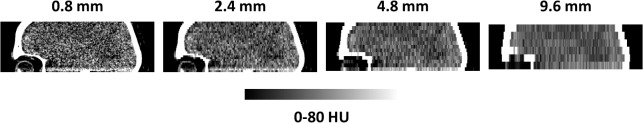
A raw CT perfusion scan at different slice thicknesses. An example of a raw, sagittal reformatted CT perfusion slice at axial slice thicknesses of 0.8, 2.4, 4.8, and 9.6 mm.

High-resolution CTP scans from 50 acute stroke patients, with an acquired axial resolution of 0.8 mm, were used to create data with slice thicknesses of 2.4, 4.8, and 9.6 mm. An example of a raw sagittal CTP slice at these four thicknesses is shown in Fig 2. To test how the measured perfusion parameters relate to the slice thickness, and how AIF resolution and motion correction affect these measurements, four different processing schemes were applied (Table 1). This resulted in 16 sets of perfusion parameter maps of which 14 are unique; the 0.8 mm resolution variations on scheme 2, 3, and 4 are the same because all schemes have a 0.8 mm AIF and thin-slice motion correction at the highest resolution.

**Table 1:** Slice thicknesses and filter types.

Scheme no.	Motion correction thickness (mm)	Filtering and analysis slice thickness (mm)	Filter type	AIF and VOF slice thickness (mm)
**1**	0.8	0.8	Gaussian	0.8
	0.8	2.4	Gaussian	2.4
	0.8	4.8	Gaussian	4.8
	0.8	9.6	Gaussian	9.6
**2**	0.8	0.8	Bilateral	0.8
	0.8	2.4	Bilateral	2.4
	0.8	4.8	Bilateral	4.8
	0.8	9.6	Bilateral	9.6
**3**	0.8	0.8	Bilateral	0.8
	0.8	2.4	Bilateral	0.8
	0.8	4.8	Bilateral	0.8
	0.8	9.6	Bilateral	0.8
**4**	0.8	0.8	Bilateral	0.8
	2.4	2.4	Bilateral	2.4
	4.8	4.8	Bilateral	4.8
	9.6	9.6	Bilateral	9.6

In scheme 1 to 3 motion correction is performed on thin slices, after which thick slices are generated. In scheme 4 motion correction is performed on thick slices.

Because the thin slice reconstructions will only be clinically acceptable if radiation dose and image noise levels do not increase, the noise level was equalized over all reconstructions by using a bilateral filter with slice thickness specific parameters [21].

### Study Design

Patients were included from the Dutch acute Stroke Trial (DUST, ClinicalTrials.gov ID: NCT00880113), of which the study protocol has been described previously [22]. Inclusion criteria for this trial were: age above 18 years, suspected ischemic stroke of less than 9 hours duration and an NIHSS score ≥2, or 1 if an indication for recombinant tissue plasminogen activator (rtPA) was present. Exclusion criteria were known renal failure or contrast allergy. The DUST study was approved by the institutional medical ethics committee (METC) and follows the ICH-GCP and WMO (Dutch act on medical research involving human subjects) guidelines and regulations. All patients or family gave signed informed consent.

From this trial database a consecutive series of 50 patients from a single center was selected to match the following additional inclusion criterion: 0.8 mm thin slice reconstructed, extended (i.e., 210 second duration) CTP acquisition on admission.

Since infarct size and location were not selection criteria, these were randomly distributed to their natural prevalence.

### Imaging Protocol

CTP was performed on admission before possible thrombolytic treatment. All included scans were acquired on a 256-slice Philips Brilliance iCT scanner (Philips Healthcare, Best, The Netherlands) at 80 kVp and 150 mAs/rotation. The scans had a total acquisition time of at least 210 seconds, divided into 25 frames with an approximately 2 second interval, a 15 second pause, and 6 frames with an approximately 30 second interval, resulting in an effective radiation dose of 3.3 mSv. At initiation of scanning, 40 mL of iopromide contrast agent (PubChem ID: 3736, Ultravist 300, Bayer HealthCare, Berlin, Germany) was injected intravenously at a rate of 6 mL/s, followed by a 40 mL saline flush.

The 0.8 mm thin slices, with a field-of view of 20 cm × 20 cm, were reconstructed in a 512 × 512 matrix using filtered backprojection. The reconstructed scans had an axial coverage of 52.0 to 64.8 mm from at least the level of the basal ganglia to the lateral ventricles to be able to assess ASPECTS levels 1 and 2 [23], resulting in 65 to 81 reconstructed slices.

### Preprocessing

Automated rigid 3D motion correction was done using the open source registration toolbox Elastix [24]. The skull served as a reference for registering all time frames to the first.

One major disadvantage of thin slice data is the increased noise level due to the increased number of voxels in the image volume. The variance of the noise will be roughly doubled when the slice thickness is halved, until it reaches the size of the point spread function of the scanner. In order to reduce the noise to an acceptable level for analysis, a filter kernel with a large enough volume is required for averaging. Although a large standard isotropic Gaussian filter can perform this task, it would introduce PVE that would nullify the resolution gained by the thinner slices. An anisotropic bilateral filter, however, is able to adapt its shape to its neighborhood and therefore average the same number of voxels with reduced PVE, whenever the neighborhood allows it [21]. In order to compare the effects of bilateral filtering to isotropic Gaussian filtering, both filters were applied (Table 1).

The values of the 3D isotropic Gaussian filter kernel *g*(**ξ**,**x**) and the 3D bilateral kernel *g*(**ξ**,**x**)*s*(**ξ**,**x**) at coordinate **ξ** are defined by

$$g\left(\xi ,x\right)=\text{exp}\;\left(-\frac{1}{2}{\left(\frac{\left\|\xi -x\right\|}{\sigma_{d}}\right)}^{2}\right)\;,\;\text{and}$$ (1)

$$s\left(\xi ,x\right)=\text{exp}\;\left(-\frac{1}{2}{\left(\frac{{\left(\bar{I}\left(\xi \right)-\bar{I}\left(x\right)\right)}^{2}}{\sigma_{r}}\right)}^{2}\right)\;,\;\text{and}$$ (2)

where **x** is the coordinate of the center voxel, *σ*_*d*_ is the spatial standard deviation (SD) of the kernel, and *σ*_*r*_ is the similarity SD. $\bar{I}\left(\xi \right)$ and $\bar{I}\left(x\right)$ are the mean CT values at coordinates **ξ** and **x** (averaged over time). The bilateral filter thus weighs both by spatial distance as well as by the squared error on the mean intensity. Both kernels require normalization.

The objective of this study was to compare perfusion parameters at different slice thicknesses but at equal noise level and dose. To this end, both the isotropic Gaussian and the anisotropic bilateral filter were adjusted for each thickness such that the SD of the noise in the filtered images, *SD*_*out*_, was reduced to a constant level of approximately 0.75 Hounsfield units (HU). For the isotropic Gaussian kernel this was achieved by using a *σ*_*d*_ of 2.5 mm for all slice0020030thicknesses. A fixed *σ*_*d*_ of 3.0 mm was used for the bilateral kernel; keeping *σ*_*d*_ fixed means that *σ*_*r*_ had to be scaled along with the slice thickness. The scaling factor *σ*_*r*_ = 3.34*SD*_*in*_ resulted in *SD*_*out*_ values of approximately 0.75. *SD*_*in*_ and *SD*_*out*_ were estimated by taking the median SD on the values in the attenuation curves within the brain tissue before the arterial bolus arrival time. The mean bolus arrival time of the data was 10.7 s (SD = 3.0 s). The filter settings and measured *SD*_*in*_ and *SD*_*out*_ values are listed in Table 2.

**Table 2:** Filter settings.

	Unfiltered	Isotropic Gaussian filter		Anisotropic bilateral filter		
Thickness (mm)	SD_in_ (HU)	σ_d_ (mm)	SD_out_ (HU)	σ_d_ (mm)	σ_r_ (HU^2^)	SD_out_ (HU)
**0.8**	14.9 (2.95)	2.5	0.76 (0.16)	3.0	50.0	0.74 (0.16)
**2.4**	11.1 (1.94)	2.5	0.75 (0.15)	3.0	37.3	0.75 (0.17)
**4.8**	8.4 (1.37)	2.5	0.77 (0.15)	3.0	28.1	0.76 (0.18)
**9.6**	6.1 (0.99)	2.5	0.70 (0.15)	3.0	20.5	0.74 (0.18)

The standard deviations *σ*_*d*_ and *σ*_*r*_ of the filter kernels, and the median noise levels of the unfiltered and filtered data, *SD*_*in*_ and *SD*_*out*_, at each slice thickness. The number between brackets is the inter-quartile range (IQR).

### Perfusion Analysis

AIFs were selected semi-automatically by searching within a manually defined circular region of interest (ROI) with a 2 cm radius for the voxel with the highest area under the curve (AUC), in keeping with the clinical standard procedure. The ROI was drawn on a 9.6 mm slice of the bilaterally filtered data, and the same ROI was applied to the 0.8, 2.4, 4.8 mm slices. Whenever possible an internal carotid artery was chosen to provide the AIF. If this location happened to be outside the imaged volume, a middle cerebral artery was chosen.

Because the axial resolution can be rather coarse with respect to the diameter of the arteries providing the AIF, the AUC of the AIF might be underestimated owing to partial volume effects. Because this leads to an overestimation of CBV, CBF, and PS, correction for partial volume is often necessary, at least in thick slice reconstructions [15,25]. The correction was provided by always selecting a VOF in the superior sagittal sinus or transverse sinus near the torcular herophili, using the same method and at the same slice thickness as the AIF, as described above.

A fast model-based non-linear regression (NLR) method was used to calculate the perfusion maps. In a previous study by our group it was shown that this NLR method not only provides the standard perfusion parameters CBV, CBF, and MTT, but also an estimate for PS, while the method is in addition insensitive to tracer delay and can be computed fast enough for application in an acute clinical setting [26].

The time frames before bolus arrival were averaged to obtain a non-contrast CT image. This non-contrast image provides the offsets for the tissue attenuation curves and it is used to segment the brain tissue. Only the voxels that had a non-contrast CT value >17 HU and <55 HU were classified as brain tissue and included in the analysis. Voxels with a blood volume >9 mL/100g were classified as vessels and excluded from the analysis. A correction factor was applied to correct the tracer concentration for the difference between the hematocrit in large vessels (AIF) and small vessels (capillaries) [27,28].

Symmetry lines were drawn manually to separate the hemispheres. The mean values of the perfusion parameters (CBV, CBF, MTT, and PS) were calculated in each hemisphere. Relative values for CBV (rCBV), CBF (rCBF), and PS (rPS) were calculated by dividing the ipsilateral means by the contralateral means, because these values are affected by scaling of the AIF. The relative MTT was not calculated as ratio, but rather as the difference in MTT (dMTT) by subtracting the contralateral mean from the ipsilateral mean, because the MTT itself is derived from the difference in width between the AIF and the tissue curves. Unlike CBV, CBF, and PS, the MTT parameter is not affected by scaling of the attenuation curves.

### Statistical Analysis

Although the noise levels of the perfusion maps were expected to be independent of the slice thickness because the noise in the filtered perfusion scans was equalized, an additional test was done to verify this hypothesis. The noise levels in the perfusion maps were estimated by dividing the 512 × 512 pixel slices into a 16 × 16 matrices of square ROIs wherein the SDs of the tissue voxels were calculated. ROIs containing less than 75% tissue voxels were excluded. The noise level was considered equal to the lowest SD found. Relative noise levels were calculated by dividing the SD by the mean perfusion value.

Statistics on the means of all absolute and relative perfusion parameters of the patient group, as well as on the AUCs of the AIF and VOF, were presented in box plots. Repeated measures ANOVA tests were applied to the perfusion measurements to test whether the mean values were significantly different for the various slice thicknesses (*P*<0.05), and to calculate the SD of the parameters due to the slice thicknesses, *s*_*B*_. The parameter *s*_*B*_^2^ is the ‘between-group variance’ as measured in an ANOVA test. It is the variance on a parameter due to the difference between slice thicknesses, disregarding the variation due to the difference between patients.

## Results

### AIF and VOF

**Fig 3:**
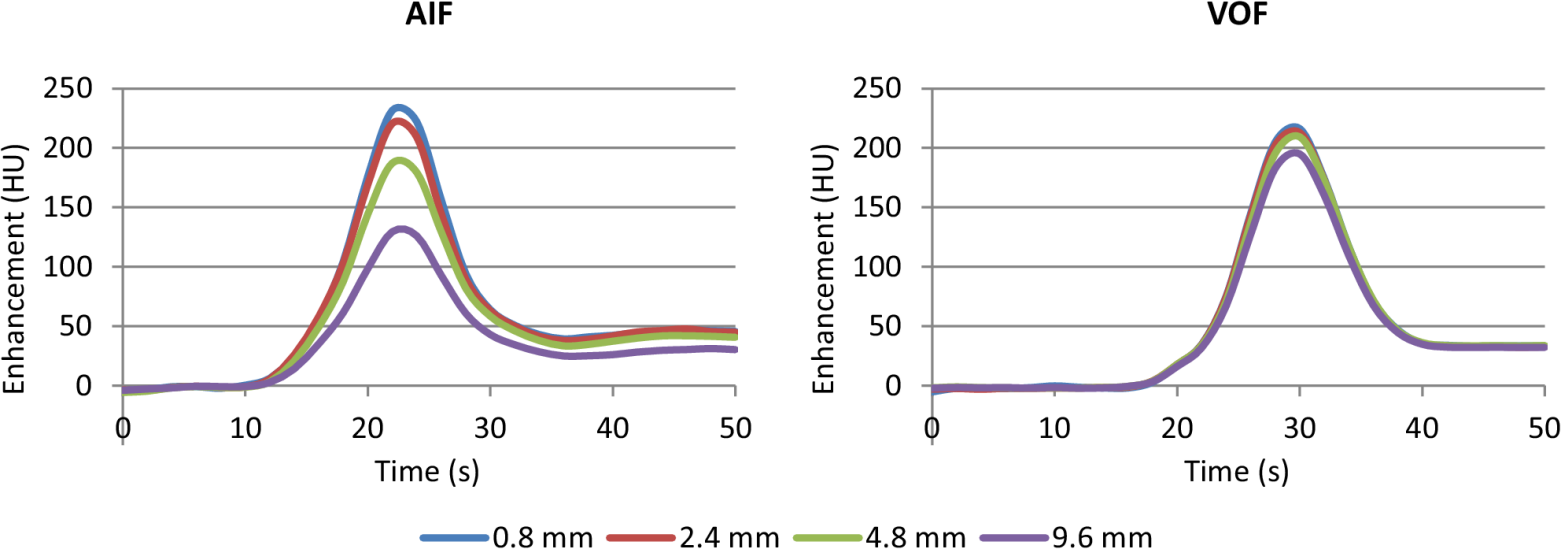
Arterial input functions and venous output functions. The mean arterial input function (AIF, left) and venous output function (VOF, right) at different slice thicknesses. Scheme 2 (see Table 1) was used for processing this data. Before averaging all AIFs and all VOFs were aligned to their time-to-peak. The areas under the curves are listed in Table 3.

An impression of the mean AIFs and VOFs is given in Fig 3. The median AUC of the VOFs, the AIF/VOF AUC ratio, and the width of the AIF as measured by gamma-variate curve fitting of the first-pass bolus are listed in Table 3. Conservation of matter requires that the AUCs of the AIF and VOF are equal in absence of partial volume effects. At a slice thickness of 0.8 and 2.4 mm the bilaterally filtered scans showed an approximately 100% AIF/VOF ratio that decreased to 69% at a slice thickness of 9.6 mm. For the isotropic Gaussian filtered scans the ratios were much lower, but the differences were less conspicuous with a median ratio of 57% at 0.8 mm thickness and 47% at 9.6 mm. Thick slice motion correction also yielded smaller AUCs and lower AIF/VOF ratios.

**Table 3:** Properties of the AIF and VOF curves.

Scheme	Thickness (mm)	AUC of VOF (HU s)	AIF/VOF AUC ratio (%)	FWHM of AIF (s)
**1—Isotropic Gaussian filter**	0.8	1627 (888)	57 (44)	10.3 (2.3)
	2.4	1576 (788)	54 (27)	10.2 (2.2)
	4.8	1492 (768)	53 (37)	10.4 (2.5)
	9.6	1394 (772)	47 (32)	10.6 (2.5)
**2—Bilateral filter**	0.8	2200 (1059)	101 (23)	9.4 (2.1)
	2.4	2129 (1086)	100 (19)	9.7 (2.1)
	4.8	2102 (1031)	91 (21)	10.0 (2.2)
	9.6	1902 (730)	69 (22)	9.9 (2.2)
**4—Thick slice motion correction**	0.8	2200 (1059)	101 (23)	9.4 (2.1)
	2.4	2120 (996)	96 (21)	9.4 (2.0)
	4.8	2068 (845)	86 (24)	9.7 (1.8)
	9.6	1893 (900)	68 (19)	10.2 (2.0)

The median (interquartile range) area under the curve of the venous output function (VOF), the ratio between the arterial input function (AIF) and VOF, and the full width at half maximum (FWHM) in case of isotropic Gaussian filtering (scheme 1), bilateral filtering (scheme 2), and bilateral filtering with thick slice motion correction (scheme 4).

The width of the AIF increased with slice thickness. The full width at half maximum (FWHM) was on average 0.5 s higher at a slice thickness of 9.6 mm as compared to 0.8 mm.

### Perfusion Maps

**Fig 4:**
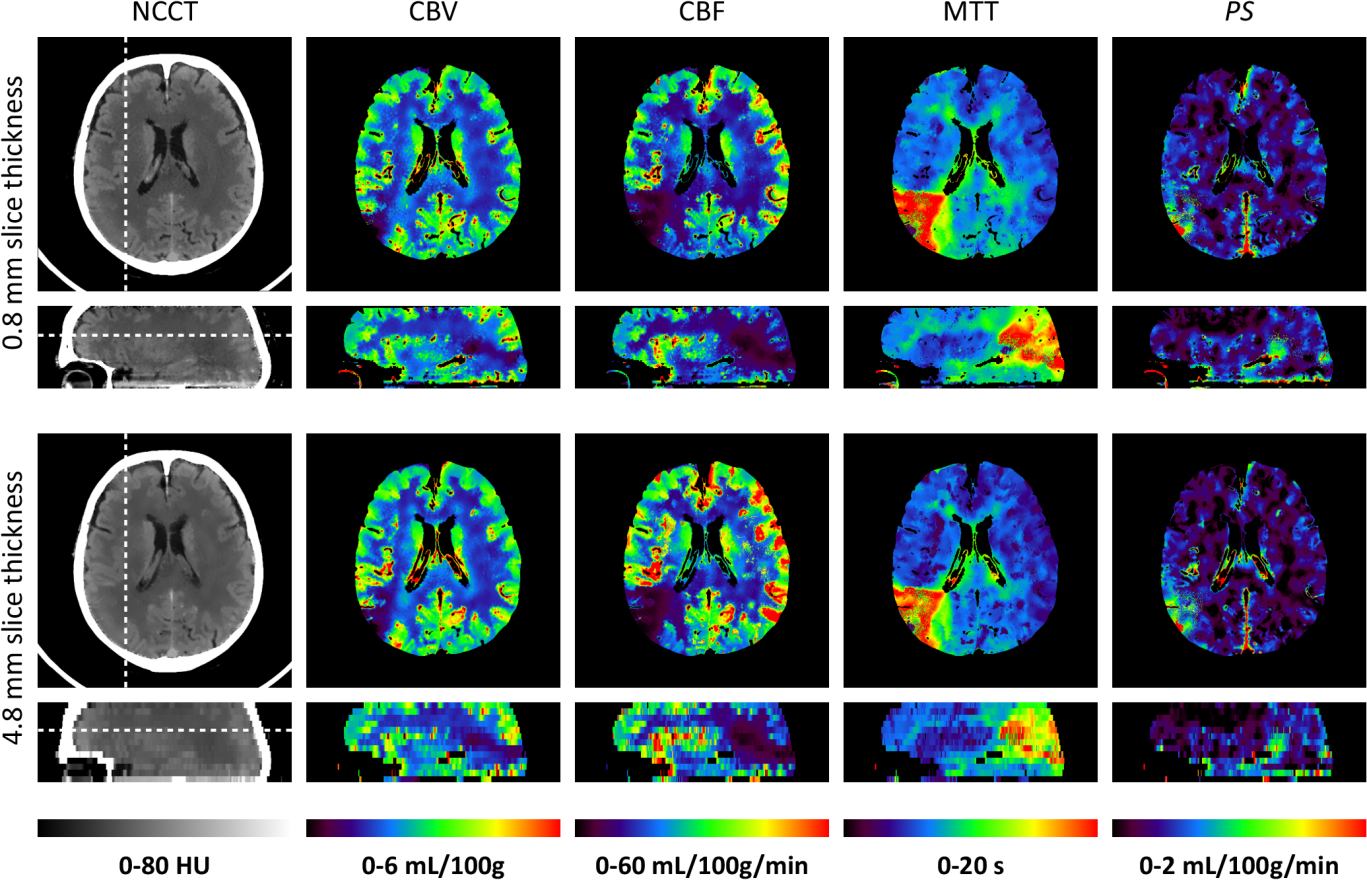
Thin and thick slice perfusion maps. An example of axial and sagittal reformatted non-contrast CT (NCCT) slices and perfusion maps with an axial slice thickness of 0.8 mm (top row) and 4.8 mm (bottom row). Scheme 2 (bilateral filtering, see Table 1) was used for processing this data. The slice positions are indicated by a dashed line in the NCCT images. The cerebral blood volume (CBV) map shows a right posterior infarct core (low blood volume), whereas the cerebral blood flow (CBF) and mean transit time (MTT) maps clearly show the surrounding penumbra (low blood flow and elevated transit time). An elevated permeability-surface area product (PS) suggests increased vascular permeability. The 4.8 mm axial slices show slightly elevated CBF and CBV values, and decreased MTT values. The 4.8 mm sagittal slices have a pixelated appearance due to the anisotropic voxels. The raw sagittal slices are shown in Fig 2.

Examples of the bilaterally filtered data and the resulting perfusion maps are shown in Fig 4. Both the CBV and the CBF map showed consistently higher values on thicker slices (bottom versus thinner slices on top row). No visible differences in noise level were noticed in either the filtered CTP scans or the perfusion maps. CBV had a median relative noise SD of 7.8% (IQR: 6.7 to 8.9%) for all 700 analyzed scans (50 patients × 14 unique schemes), CBF of 12.0% (IQR: 9.3 to 14.8%), MTT of 7.8% (IQR: 5.6 to 10.7%), and PS of 5.6% (IQR: 2.8 to 10.0%).

### Absolute Measurements

**Fig 5:**
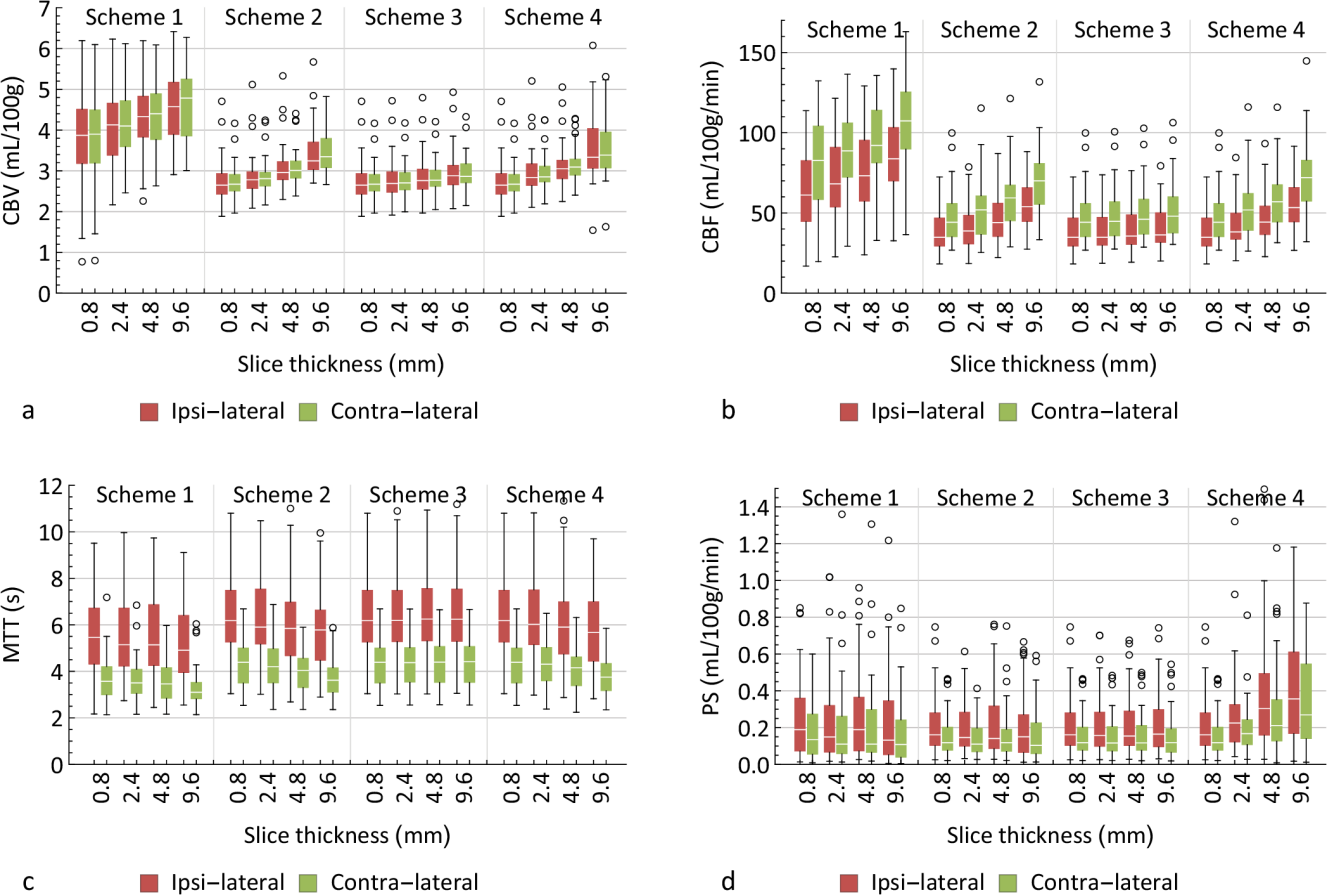
Absolute perfusion values in the ipsilateral and contralateral hemispheres. Box plots showing the absolute perfusion values that were measured in the tissue within the ipsilateral hemisphere (red) and in the contralateral hemisphere (green). Outliers, marked by circles, are defined as points that are more than 1.5× the interquartile range above the 75% quartile or below the 25% quartile.

The distributions of the perfusion values (mean of the tissue voxels in both hemispheres) are shown in the box plots in Fig 5. Table 4 lists the mean values of the absolute perfusion parameters, the standard deviations due to slice thickness, and *P*-values of the effect of slice thickness on the parameters.

**Table 4:** Mean and variation in absolute perfusion parameters.

		Scheme 1			Scheme 2			Scheme 3			Scheme 4		
Parameter	Side	Mean	s_B_	P	Mean	s_B_	P	Mean	s_B_	P	Mean	s_B_	P
**CBV**	**Ipsilateral**	4.2	2.3	0	3.0	2.3	0	2.8	0.72	0	3.1	2.5	0
	**Contralateral**	4.2	2.4	0	3.0	2.2	0	2.8	0.66	0	3.1	2.5	0
**CBF**	**Ipsilateral**	74	62	0	45	53	0	39	7.9	0	46	56	0
	**Contralateral**	92	75	0	57	68	0	49	11	0	58	73	0
**MTT**	**Ipsilateral**	5.4	1.1	0	6.2	2.0	0	6.4	0.17	0	6.2	1.9	0
	**Contralateral**	3.5	1.2	0	4.1	1.9	0	4.3	0.11	0	4.1	1.8	0
**PS**	**Ipsilateral**	0.28	0.23	0.37	0.24	0.34	0.21	0.32	0.05	0.12	0.36	0.49	0.09
	**Contralateral**	0.21	0.23	0.32	0.18	0.31	0.11	0.25	0.04	0.19	0.27	0.39	0.07

The means, standard deviations (*s*_*B*_), and *P*-values of the parameters, compared between slice thicknesses (repeated measures ANOVA). The parameters are subsequently cerebral blood volume (CBV, in mL/100g), cerebral blood flow (CBF, in mL/100g/min), mean transit time (MTT, in s), and permeability-surface area (PS, in mL/100g/min). The s_B_ is the standard deviation on a parameter due to the difference between slice thicknesses, disregarding the variation due to the difference between patients. A parameter with a *P*-value <0.05 was considered significantly affected by the slice thicknesses. *P*-values <0.01 are shown as 0.

Absolute CBV and CBF values were higher with isotropic Gaussian filtering than with bilateral filtering (Fig 5A and 5B), whereas MTT values were a little lower (Fig 5C).

In all schemes, the absolute values of CBV, CBF, and MTT differed significantly between slice thickness (*P*<<0.05). Fig 5A and 5B show positive trends for CBV and CBF with increasing slice thickness, and a slight negative trend in MTT. The variances due to slice thickness were much smaller in scheme 3 than in the other schemes. Absolute CBF and CBV were 22% and 14% lower on 0.8 mm as compared to 4.8 mm slices in scheme 4, but these differences were reduced to just 3% and 4% in scheme 3.

Although the box plot for absolute PS seems to show a trend in scheme 4 (Fig 5D), the absolute PS values did not significantly differ as a function of slice thickness in any case. Absolute PS values were 46% lower on 0.8 mm than on 4.8 mm slices in scheme 4, but these differences were reduced to just 3% in scheme 3.

### Relative Measurements

Table 5 lists the mean values of the relative perfusion parameters, the standard deviations due to slice thickness, and *P*-values of the effect of slice thickness on the parameters. Differences between schemes, but also differences within schemes due to slice thickness were much smaller than for the absolute values.

**Table 5:** Mean and variation in relative perfusion parameters.

	Scheme 1			Scheme 2			Scheme 3			Scheme 4		
Parameter	Mean	s_B_	P	Mean	s_B_	P	Mean	s_B_	P	Mean	s_B_	P
**rCBV**	99	2.1	0.15	100	1.3	0.21	100	2.3	0	100	0.59	0.90
**rCBF**	81	2.3	0.33	80	1.1	0.70	80	1.0	0.17	80	0.84	0.89
**dMTT**	1.6	0.22	0	1.5	0.20	0	1.5	0.005	0.98	1.5	0.20	0
**rPS**	149	72	0	144	42	0.01	143	28	0.02	146	79	0.06

The means, standard deviations (*s*_*B*_), and *P*-values of the parameters, compared between slice thicknesses (repeated measures ANOVA). The parameters are subsequently relative cerebral blood volume (rCBV, in %), relative cerebral blood flow (rCBF, in %), difference in mean transit time (dMTT, in s), and relative permeability-surface area (rPS, in %). The s_B_ is the standard deviation on a parameter due to the difference between slice thicknesses, disregarding the variation due to the difference between patients. A parameter with a *P*-value <0.05 was considered significantly affected by the slice thicknesses. *P*-values <0.01 are shown as 0.

Although *s*_*B*_ values were small, rCBV was significantly affected by the slice thickness in scheme 3 (*P*<0.05). dMTT was also significantly different in scheme 1, 2, and 4, just as rPS in scheme 1, 2, and 3. In all these cases increasing slice thickness gave a small but significant positive bias to rCBV, dMTT, as well as to rPS. Because the interpatient variability is much larger than these biases, they cannot be observed in Fig 5.

Stroke patients with an early infarct usually have a small infarct core, thus a small CBV defect on CTP imaging. For this reason the mean rCBV values were close to 100%. The volume of the surrounding penumbra, showing reduced CBF without reduced CBV, is usually much larger; mean CBF values were 20% lower, mean MTT values were 1.5 s higher, and mean PS values were 46% higher in the ipsi-lateral hemisphere.

## Discussion

The results of this study show how the use of thin slice CTP data for stroke diagnosis at equal noise level and radiation dose may be feasible without affecting relative perfusion values. It might however require revision of current clinical thresholds on absolute perfusion values. Partial volume effects (PVE) in the AIF plays a key role. Because even large veins are affected by PVE, scaling of the AIF to match the AUC of the VOF does not fully solve this issue. If the same PVE-free (thin slice) AIF is used for analysis of all slice thicknesses, slice thickness is of minor influence on the absolute perfusion values.

It was found that the PVE in the AIF and VOF may explain most, if not all findings in this study. PVE not only decreases the AUC of the AIF and VOF, but also increases the width of the AIF. For this reason PVE influences all perfusion parameters, including the MTT. PVE in tissue TACs only marginally influences the perfusion values, since the effect of partial volume on relatively large, homogeneous tissue regions is smaller than the PVE in vessels.

Both slice thickness and filtering methods influence the amount of PVE. Axel already described that CTP analysis would require venous attenuation curves to correct for partial volume in the AIF [25]. As expected, this study also showed that the AUC of the AIF reduces with increasing slice thickness. In line with a previous study by our group [15], it can be concluded that a slice thickness of approximately 2 mm is required to measure a PVE free AIF in the internal carotid artery (roughly 3.5 to 5 mm in diameter [29,30]) or in the middle cerebral artery (2.5 to 4 mm in diameter). Even the VOF, which is measured in much larger veins and used to rescale the AIF, is affected. Although bilateral filtering greatly reduces PVE as compared to isotropic Gaussian filtering (Table 3), the AUC of the VOF is on average as much as 13.5% lower at a slice thickness of 9.6 mm than at 0.8 mm.

Absolute CBV and CBF values were found to be higher with isotropic Gaussian filtering than with bilateral filtering. Because each AIF was scaled to match the AUC of the VOF, this is most likely caused by the difference in AUCs of the VOFs between both filters. The MTT values were also found to be a little lower with Gaussian filtering, which is probably caused by widening of the AIF due to PVE.

The effect of slice thickness on the PVE in the VOF and therefore on the perfusion values is most pronounced when the CBV and CBF values in scheme 3 are compared with the values in other schemes. Because of the use of the PVE-free (thin slice) AIFs and VOFs on all slice thicknesses, the values in scheme 3 are similar between slice thicknesses, whereas the other schemes clearly showed a positive trend for these parameters. This trend is in line with what was found by Van der Schaaf et al. [20].

A small negative trend was observed for the absolute MTT. Just like the use of the isotropic Gaussian filter resulted in lower MTT values, an increase in slice thickness also decreased MTT. As the MTT values in scheme 3 did not show this trend, the trend is most likely caused by widening of the AIF due to PVE, as shown in Table 3.

Although the median PS was not found to be affected by slice thickness, a positive trend was observed in scheme 4 (Fig 5D). It is very likely that this trend is not caused by PVE, but rather by the errors introduced by motion artifacts. These errors give a positive bias to PS values, resulting in an apparent positive trend [26]. PS values are probably more sensitive to motion artifacts than other perfusion parameters because they are influenced by the time frames measured in the delayed phase (after the first pass bolus). In a longer time frame the chance of motion induced errors increases. When motion correction was performed on thin slices, as in schemes 1, 2, and 3, the trend disappeared.

In contrast to the finding that absolute quantification is mostly hindered by PVE in large arteries and veins, it was found that relative perfusion values were nearly unbiased. Scaling errors of CBV and CBF due to PVE in the AIF and VOF had no clinically relevant effect on their ipsi-lateral versus contra-lateral ratios, i.e. rCBV and rCBF. Similarly, MTT values in both hemispheres were equally biased due to widening of the AIF, causing the dMTT to be nearly unbiased. The cause of these small, but statistically significant biases is unknown. It should however be noted that the observation of a statistically significant bias does not necessarily imply a relevant effect, but it may merely indicate a precise measurement with some small systematic drift.

The data showed that the spread in absolute values between patients is quite large, especially for the CBF, MTT, and PS parameters. Although there might be significant interpatient variability, these parameters are also fundamentally more difficult to estimate than CBV. Whereas CBV is estimated by comparing AUCs, estimating CBF and MTT requires high-frequency information which is to a very limited extent available in the relatively smooth and wide AIF. The PS parameter is a very weak component of the tissue curves, requiring high quality curves, free of motion, over a long scan duration. When it is not possible to use a high-resolution, PVE-free AIF, then the use of relative perfusion values is advisable.

Further study is required to find how the assumed advantages of using thin slice data, for example improved inter-observer and intra-observer variability or the detection of lacunar infarcts, work out in clinical practice.

## Conclusions

Performing thin slice CTP analysis at equal noise level and radiation dose might be feasible without affecting relative perfusion values. Due to PVE in large arteries and veins, it does however affect absolute perfusion values, of which CBF and CBV are most sensitive. Bilateral filtering enables the analysis of thin slice data, which may be used to more accurately locate and delineate the infarct core and penumbra and estimate blood-brain barrier permeability in CT perfusion imaging of acute ischemic stroke. Using thin slice data enables a PVE-free AIF selection for perfusion analysis, which gave the least variance in both absolute and relative perfusion values between slice thicknesses. In addition, motion artifacts can be reduced most effectively by 3D motion correction on thin slice data. Since the current thresholds on absolute values are generally established using AIFs and VOFs measured in scans with a slice thickness of 5 to 10 mm, they cannot be applied to thin slice data and may therefore need to be revised.
